# Phylogeny and evolution of life-history strategies in the Sycophaginae non-pollinating fig wasps (Hymenoptera, Chalcidoidea)

**DOI:** 10.1186/1471-2148-11-178

**Published:** 2011-06-22

**Authors:** Astrid Cruaud, Roula Jabbour-Zahab, Gwenaëlle Genson, Finn Kjellberg, Noppol Kobmoo, Simon van Noort, Yang Da-Rong, Peng Yan-Qiong, Rosichon Ubaidillah, Paul E Hanson, Otilene Santos-Mattos, Fernando HA Farache, Rodrigo AS Pereira, Carole Kerdelhué, Jean-Yves Rasplus

**Affiliations:** 1 INRA-UMR Centre de Biologie et de Gestion des Populations, CBGP, (INRA/IRD/CIRAD/Montpellier SupAgro), Campus international de Baillarguet, CS 30016, 34988 Montferrier-sur Lez, France; 2 CNRS-UMR Centre d'Ecologie Fonctionnelle et Evolutive, CEFE, 1919 route de Mende, 34293 Montpellier Cedex 5, France; 3 Natural History Division, South African Museum, Iziko Museums of Cape Town, PO Box 61, Cape Town 8000, South Africa; 4 Key Laboratory of Tropical Forest Ecology, Xishuangbanna Tropical Botanical Garden, Chinese Academy of Sciences, 88 Xuefu Road, 650223 Kunming, Yunnan, China; 5 Entomology Laboratory, Zoology Division (Museum Zoologicum Bogoriense). Center Research for Biology, LIPI, Gedung Widyasatwaloka Jl. Raya Jakarta-Bogor, Km 46, Cobinong 16911, Bogor, Indonesia; 6 Escuela de Biología. Universidad de Costa Rica. A.P. 2060 San Pedro de Montes de Oca. San José, Costa Rica; 7 Instituto Nacional de Pesquisa da Amazônia, av Andre Araujo 2936, 69060-001, Manaus, Amazonas, Brazil; 8 Depto de Biologia/FFCLRP-USP, Av. Bandeirantes, 3900, 14040-901 - Ribeirão Preto, SP, Brazil

## Abstract

**Background:**

Non-pollinating Sycophaginae (Hymenoptera, Chalcidoidea) form small communities within *Urostigma *and *Sycomorus *fig trees. The species show differences in galling habits and exhibit apterous, winged or dimorphic males. The large gall inducers oviposit early in syconium development and lay few eggs; the small gall inducers lay more eggs soon after pollination; the ostiolar gall-inducers enter the syconium to oviposit and the cleptoparasites oviposit in galls induced by other fig wasps. The systematics of the group remains unclear and only one phylogeny based on limited sampling has been published to date. Here we present an expanded phylogeny for sycophagine fig wasps including about 1.5 times the number of described species. We sequenced mitochondrial and nuclear markers (4.2 kb) on 73 species and 145 individuals and conducted maximum likelihood and Bayesian phylogenetic analyses. We then used this phylogeny to reconstruct the evolution of Sycophaginae life-history strategies and test if the presence of winged males and small brood size may be correlated.

**Results:**

The resulting trees are well resolved and strongly supported. With the exception of *Apocrytophagus*, which is paraphyletic with respect to *Sycophaga*, all genera are monophyletic. The Sycophaginae are divided into three clades: (i) *Eukoebelea*; (ii) *Pseudidarnes*, *Anidarnes *and *Conidarnes *and (iii) *Apocryptophagus*, *Sycophaga *and *Idarnes*. The ancestral states for galling habits and male morphology remain ambiguous and our reconstructions show that the two traits are evolutionary labile.

**Conclusions:**

The three main clades could be considered as tribes and we list some morphological characters that define them. The same biologies re-evolved several times independently, which make Sycophaginae an interesting model to test predictions on what factors will canalize the evolution of a particular biology. The ostiolar gall-inducers are the only monophyletic group. In 15 Myr, they evolved several morphological adaptations to enter the syconia that make them strongly divergent from their sister taxa. Sycophaginae appears to be another example where sexual selection on male mating opportunities favored winged males in species with small broods and wingless males in species with large broods. However, some species are exceptional in that they lay few eggs but exhibit apterous males, which we hypothesize could be due to other selective pressures selecting against the re-appearance of winged morphs.

## Background

In many animal species, males that compete for females may adopt alternative reproductive tactics. These tactics translate in many cases into discontinuous variation in male morphology, behaviour or life history [1]. Male dimorphism or polymorphism is common in species in which sexual selection is strong [2,3] and takes place in a variety of taxonomic groups such as insects (e.g. bees and wasps [4,5], damselflies [6], earwigs [7], dung-beetles [8]); other invertebrates (e.g. spiders [9], opiliones [10], mites [11], amphipods [12] and nematodes [13]) and vertebrates [14,15].

Among these groups, fig wasp is a well known model to study male polymorphism [16-25]. Intense sexual selection in fig wasps has lead to competing males evolving exaggerated morphologies to mate, thereby enhancing their reproductive success [26]. Beside winged males that tend to look similar to winged females, wingless males of fig wasps exhibit at least five different morphs [27]. In some cases males are so different from females that they were initially described as different species or even genera [28]. Wingless males engage in lethal combat for access to females [16,29,30], and consequently exhibit also considerable morphological diversity [20,25], whereas winged males disperse to mate with females outside the syconium.

Fig wasps are a polyphyletic assemblage of Chalcidoidea (and few braconids) that develop in the inflorescences of fig trees (Moraceae, *Ficus*). They are subdivided into pollinating fig wasps that belong to the family Agaonidae (but see [31,32]) and non-pollinating fig wasps (NPFW) that comprise, among others, five unrelated subfamilies strictly associated with *Ficus*: Otitesellinae, Sycoecinae, Sycoryctinae, Epichrysomallinae and Sycophaginae [33].

NPFW are associated with almost all the *ca *750 *Ficus *species worldwide. They form rich communities of interacting species (up to 36) that differ among regions of the world and among groups of *Ficus*. The genus *Ficus *is characterized by its unique enclosed inflorescence, the syconium, which is an urn-shaped receptacle that contains tens to thousands flowers. Most species of NPFW oviposit from outside the syconium but some species enter receptive syconia through a narrow tunnel called the ostiole and oviposit in the flowers from inside the syconium. The biology of NPFW are poorly known and few detailed studies have reported reliable observations. Among these five groups of fig wasps, the subfamily Sycophaginae stand out as the most diverse in terms of male morphologies, biology and galling habits as well as timing of oviposition.

### Taxonomy, diversity and distribution of sycophagine fig wasps

For a long time, Sycophaginae was considered to be the only subfamily of NPFW [33-35]. The classification and the taxonomic limits of the group have been modified several times. Sycophaginae have been classified as a subfamily of Torymidae [35-38], or of Agaonidae [33]. Presently, they are not assigned to a family [39] and their phylogenetic position within Chalcidoidea remains unknown [40].

**Figure 1:**
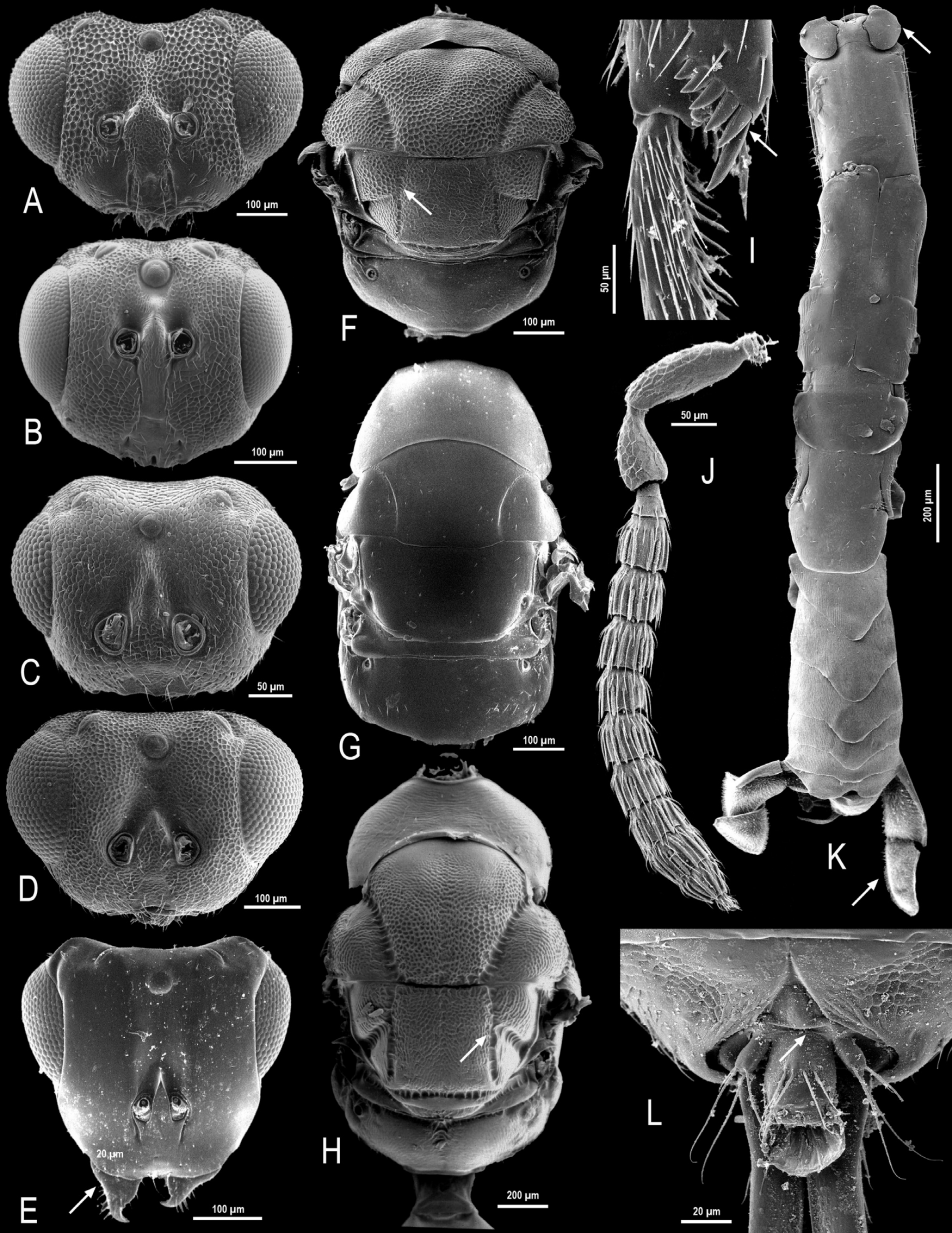
**Morphological features of the Sycophaginae**. Mesosoma female: A. *Eukoebelea*, B. *Apocryptophagus*. D *Anidarnes*. E. *Sycophaga*. Head female: G. *Apocryptophagus*, H. *Anidarnes*, J. *Sycophaga*, K. *Eukoebelea*. Tibial spurs female *Sycophaga*: C. Fore leg, F. Hind leg. Dorsal habitus of male. I. *Apocryptophagus*. Tergum 8 and epipygium female. L. *Apocryptophagus*.

The subfamily Sycophaginae, in its modern sense, is well defined by two apomorphies: 1) the presence of grooves framing the scutellum (precisely the scutellar-axillar complex bears straight or incurved axillular grooves and transverse frenal grooves, Figure 1A, B &1D) [41]; 2) the structure of the gastral tergite 8 (exhibiting a posterior margin deeply, sinuately A-like, with small, sclerotized, thumbnail-like medial flap (epipygium) and with a peg-like cercus arising from the membrane on either side of the epipygium, Figure 1L) [40].

**Figure 2:**
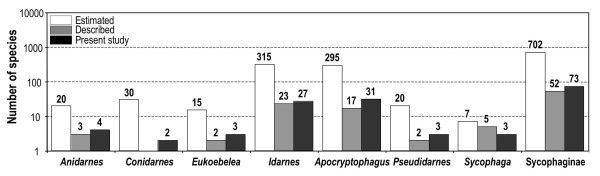
**Number of species of Sycophaginae estimated, described and included in the present study (logarithmic scale)**.

Presently, Sycophaginae comprises 6 genera and 52 described species (Figure 2, Table 1): *Anidarnes *Bouček (3 described species), *Apocryptophagus *Ashmead (19), *Eukoebelea *Ashmead (2), *Idarnes *Walker (22), *Pseudidarnes *Girault (2) and *Sycophaga *Westwood (4). A new genus, associated with fig trees of section *Conosycea *in the Oriental region has recently been discovered by A.C. and J.Y.R. Hereafter, this new genus will be referred as *Conidarnes nomem provis*. *Idarnes *is subdivided into three species-groups, one of which (*incerta *species group) strongly differs morphologically from the others [42,43].

**Table 1:** Taxonomy, distribution and life-history stategies of sycophagine fig wasps.

Genera	Host *Ficus *subgenus (sub)section	Distribution	Oviposition timing	Ovipositor length	Gall size	Male morphology
*Anidarnes*	*Urostigma Americana*	Neotropical	Before pollination (group 1)	shorter than body length	Large	Winged
*Eukoebelea*	*Urostigma Malvanthera*	Australasian	After pollination (group 5)	equal or longer than body length	Small	Winged/Wingless
*Conidarnes*	*Urostigma *Conosycea	Oriental	Before & during pollination (groups 1 & 3)	shorter than body length	Large	Winged
*Idarnes *group *incerta*	*Urostigma Americana*	Neotropical	Before pollination (group 1)	shorter than body length	Large	Winged
*Idarnes *group *flavicollis*	*Urostigma Americana*	Neotropical	After pollination (group 2)	longer than body length	Small	Wingless
*Idarnes *group *carme*	*Urostigma Americana*	Neotropical	After pollination (group 4)	longer than body length	Small	Wingless
*Apocryptophagus*	*Sycomorus*&*Urostigma Urostigma*	Afrotropical + Oriental + Australasian	Before & After pollination (groups 1, 2, 4 & 5)	Shorter, equal to or longer than body length	Small	Wingless
*Pseudidarnes*	*Urostigma Malvanthera*	Australasian	Before pollination (group 1)	shorter than body length	Large	Winged
*Sycophaga*	*Sycomorus*	Afrotropical	During pollination (group 3)	shorter than body length	Small	Wingless

Extrapolating from our sampling of several hundred species of *Ficus *species the total diversity of the Sycophaginae could reach 700 species worldwide. Consequently, more than 80% of the species of Sycophaginae probably await description (Figure 2). The subfamily occurs in all tropical regions of the world and is associated with two unrelated subgenera of *Ficus*, namely *Urostigma *and *Sycomorus *[44,45] (Table 1). More precisely, Neotropical *Idarnes *and *Anidarnes *wasps develop in *Ficus *of section *Americana *(subgenus *Urostigma*), Australasian *Eukoebelea *are strictly associated with *Ficus *section *Malvanthera *(subgenus *Urostigma*), Oriental *Conidarnes *are associated with section *Conosycea *(subgenus *Urostigma*); while *Apocryptophagus *and *Sycophaga *species are associated with the fig trees of the subgenus *Sycomorus*, with the exception of two species of *Apocryptophagus *found on *F. orthoneura *(subgenus *Urostigma*, section *Urostigma*) in Southern China during this study.

Only a few species-level phylogenies of Sycophaginae have been published so far, mostly on Papuan *Apocryptophagus *[46,47] and Neotropical *Idarnes *[48]. A recent study by Cruaud *et al*. [45] reconstructed the historical biogeography of Sycophaginae and proposed a first hypothesis based on 55 species. However, the authors focused neither on the taxonomy of the group nor on the evolution of the life-history strategies.

### Biology of sycophagine fig wasps

Sycophaginae use chemical mediation to locate fig trees [49]. Up to 6 species of Sycophaginae can be found on the same *Ficus *species [50-55]. In most cases, these small communities are structured by the development of the syconium, the timing of oviposition (before, during or after pollination) and the feeding habits of the wasps (gallers vs. cleptoparasites) [50,51,56]. The biology of sycophagine NPFW remains poorly known and extremely difficult to ascertain. For some species, trophic status is inferred by oviposition behaviour and biology remains suspected.

**Figure 3:**
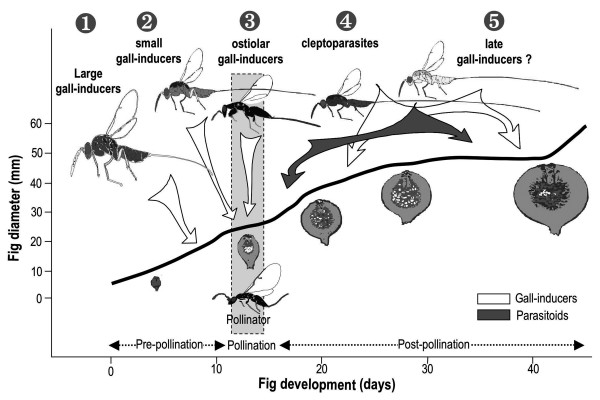
**Ecological groups of sycophagine NPFW**. The five ecological groups are depicted on the growth curve of a *Sycomorus *fig. The arrows show the timing of oviposition of the different ecological groups of Sycophaginae.

Five different ecological groups of species can be recognized [57,58] (Table 1 Figure 3). Hereafter, the size of the wasps in defining the groups is always given relative to the size of the other fig wasp species, including the pollinator(s), associated with the same species of *Ficus*. This is also true for the length of the ovipositor which is given relative to the ovipositor length of the other fig wasp species associated with this specific *Ficus *(ie longer, shorter or same length; measurements taken with a reticule under a microscope). Consequently, a large gall-inducer within one fig wasp guild (associated to one fig tree species) could be smaller than the smaller fig wasp associated with another *Ficus *species. Even if few exceptions occur, the size of the fig wasps associated with one *Ficus *species is globally correlated to the size of the syconium, the smaller the syconium is, the smaller the flowers and the fig wasps are.

#### Group 1. The large gall-inducers

This group contains wasps much larger than the co-occurring pollinators. They exhibit relatively shorter and thicker ovipositor (shorter than their body length) than other Sycophaginae ovipositing in the same syconia. They oviposit through the syconium wall seven to 12 days before pollination (data on one species of the *Idarnes incerta *species group [19] and two species of *Apocryptophagus *[21,59]). The number of galls per syconium is variable [60]. However, the published data showed that there is a good correlation between large galls and small brood size (i.e. the mean number of conspecific sycophagine wasps developing per syconium [61-63]).

The large gall-inducers induce large galls that protrude into the syconium cavity and can occupy nearly the entire volume of the syconium [63]. There is no documentation of any such large wasps possessing an ovipositor as long as that of small gall-inducers ovipositing on the same syconium. This suggests that this group is biologically homogeneous. This ecology occurs within several genera: *Anidarnes *(all species), *Conidarnes *(most), *Pseudidarnes *(all), *Idarnes incerta *species group (all) and *Apocryptophagus *(a few).

#### Group 2. The small gall-inducers

These smaller wasps (about the same size than the co-occuring pollinators), exhibit medium to long and thin ovipositor (longer than body length). They oviposit into the fig flowers a few days before fig pollination (direct demonstration for *A. testacea *[23]) or during fig pollination (direct demonstration for *A. fusca *[23]) and induce galls about the same size as those of the pollinator. At least one species of *Idarnes *is known to insert its ovipositor through the stigma and the style, and deposit its eggs between the inner integument and the nucellus [50], in this sense their oviposition is similar to that of the pollinators (although they oviposit from the outside). This type of gall-inducer occurs in many syconia at medium to high numbers and there are often many species of wasps per fig tree species. Available data on this group are still scarce but on average their brood sizes are medium to large [52,54,62,64], although some species appear to have relatively small brood size (e.g. *Apocryptophagus fusca *[54]. These species frequently exhibit different ovipositor lengths relative to their body size [65]. They belong to the genus *Apocryptophagus *(most, experimental demonstration for two species [23]) and to the *Idarnes flavicollis *group (possibly all, experimental demonstration for one species [19]). Experimental demonstration consisted of introductions of females into bagged syconia and determining if wasps could develop independently of the presence of other fig wasps. This experiments ascertained the ability of these sycophagine wasps to induce galls.

#### Group 3. The ostiolar gall-inducers

This group contains species - about the same size as the co-occurring pollinators - that enter the syconia through the ostiole to oviposit, just like the pollinators do. *Sycophaga sycomori *drives its ovipositor along and not through the style of the flower (short or long-styled) and generally deposits one egg into the embryo sac [66]. They frequently occur in the syconia and have relatively large brood sizes [52,66]. The Afrotropical genus *Sycophaga *and possibly also one undescribed oriental species of *Conidarnes *(JYR, pers. obs.) belong to this group. Ostiolar gallers are apparently absent from the Neotropics.

#### Group 4. The cleptoparasites

In the fig wasp literature the terms "inquiline" [67] and "cleptoparasite" [57,68] are sometimes used loosely as synonyms. Here we follow Gordh and Headrick (2001) [69] who defined the two words differently: an inquiline is a species that lives as a "guest" of another species but does not harm the host species. This happens in Cynipid wasps in which the development of the inquiline in a host gall is not always lethal to the gall inducer [70]. A cleptoparasite is a parasite that preferentially attacks a gall already parasitized by another species of parasite. Consequently, cleptoparasites have always strong detrimental effect on the reproductive success of their host. Here we use the term "cleptoparasites" to refer to several groups of Sycophaginae for which detrimental effects on the reproductive success of the pollinators have been observed [52,53]. As we have no direct evidence of sycophagine species developing as a strict parasite of another wasp larvae, the term "parasitoid" is not used here.

This fourth group is composed of smaller wasps (about the size of the co-occurring pollinators) that exhibit relatively longer ovipositor compared to other Sycophaginae occurring in the same fig trees (in average the ovipositor is much longer than the body length). They lay eggs one to three weeks after pollination (*Idarnes*, [50]). In the few documented cases, these wasps oviposit in galls induced by other fig wasp species and containing mostly larva of the pollinator (sometimes also of NPFW) [54,65,67]. They insert their ovipositor through the gall pericarp (*Idarnes carme *group), deposit their egg inside the embryo sac and the larva consumes the endosperm as well as the gall-maker larva [50]. These species have relatively large brood size [64]. At least in one species, when the parasitic pressure is high, the wasps (*Idarnes*) can oviposit in developing seeds and produce small males [67]. These wasps belong to the genus *Apocryptophagus *(some, direct demonstration for *A. agraensis *[23]) and to *Idarnes carme *group (all, observation of timing of oviposition for one species [19]).

#### Group 5. The late "gall-inducers"

Some species groups generally recognized as gall-inducers oviposit after fig pollination. It is difficult to understand how these wasps that oviposit well after pollination could be gall-inducers. Indeed female flowers wither rapidly after pollination [24] suggesting that they can no longer be galled. Further, space constraints due to the tight internal flower packing resulting from ovule growth following pollination, probably prohibit new galls from developing [19]. Hence these species are probably cleptoparasites, parasitoids of the gall-maker larva or even hyperparasitoids [71]. They are abundant and, for the few studies published, seem to have relatively large brood size [52]. Nevertheless, for conservative reasons we will call them late gall-inducers, pending further biological data. The members of this group belong to the genera *Eukoebelea *(all ?) and *Apocryptophagus *(some species [20]).

### Male wingedness

Sycophaginae exhibit extreme male polymorphism (Table 1). Some species-groups or genera have only winged males (*Idarnes incerta *group, *Anidarnes*, *Conidarnes*), some have dimorphic males (both winged and apterous individuals co-occur in the same syconia, *e.g*. *Eukoebelea *and *Pseudidarnes*), whereas the others have only wingless males (*Sycophaga*, *Apocryptophagus*, other groups of *Idarnes*).

Hamilton [72] proposed that sexual selection on male mating opportunities favored winged males in NPFW species with small broods and wingless males in species with large broods. Indeed, in species with large broods, females develop in syconia with conspecific males and mate before dispersal. Consequently, most mating opportunities are preempted by the apterous males particularly adapted to crawl between the flowers and mate within the syconium cavity. In contrast, in species with small broods, most females emerge into the syconium cavities in the absence of conspecific males, and winged males that are able to leave their natal syconium enjoy most mating opportunities [61].

### Aims of the study

As mentioned above, sycophagine are ecologically diverse and show some of the most extreme examples of male dimorphism among fig wasps. Therefore, they appear to be good candidates to test Hamilton's hypothesis predicting that the presence of winged males and small brood size may be correlated. In this paper we employ multiple genetic loci, extensive taxon sampling over all known genera, and several different analytical approaches to discuss the generic delimitation and reconstruct the evolutionary history of sycophagine fig wasps. We then use this phylogeny to reconstruct the evolution of sycophagine life-history strategies (galling habits/brood size and male polymorphism) based on information compiled from our own observations and several literature sources [50-54,60,61,63,65,73-76]. In this context, we propose hypotheses that could explain the observed male dimorphism within sycophagine fig wasps.

## Results

### Sequence data

The final matrix contained 73 ingroup species and 4 outgroups, represented by 145 individuals and 4267 bp (*COI*+*Cytb *= 2231 bp, *EF *= 516 bp, 28S stems = 933 bp, 28S loops = 587 bp). Of these, 2307 bp were variable and 1984 bp were parsimony informative. Alignment of exons revealed no indels. For all partitions the GTR+I+G was determined as the best-fitting model by MrAIC.

### Phylogenetic analysis

**Figure 4:**
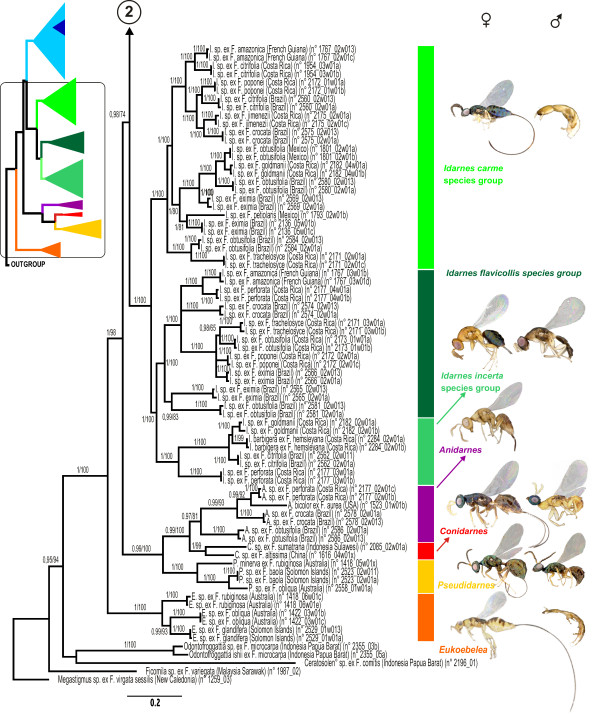
**Phylogram of relationships among Sycophaginae and the five outgroup taxa**. Bayesian posterior probabilities ≥ 0.95 and likelihood bootstrap values ≥ 65 are indicated above branches.

**Figure 5:**
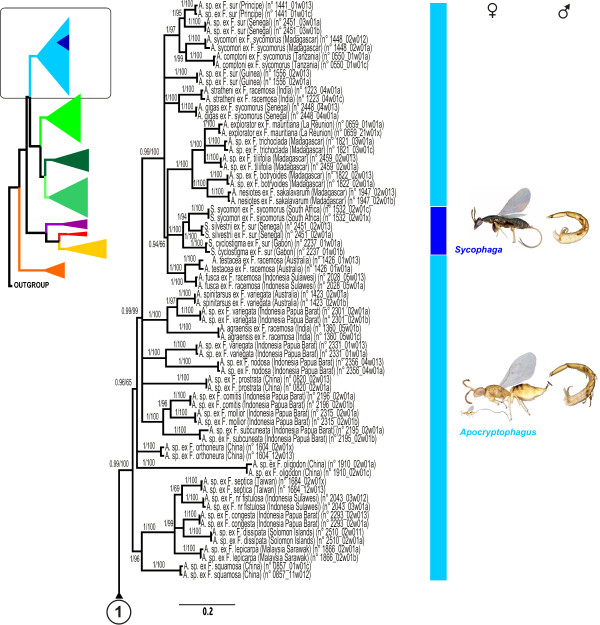
**Phylogram of relationships among Sycophaginae and the five outgroup taxa (continued)**. Bayesian posterior probabilities ≥ 0.95 and likelihood bootstrap values ≥ 65 are indicated above branches.

ML and Bayesian analyses produced similar topologies. We chose to map node support values (PP and BP) on the Bayesian topology (Figures 4, 5). The topology was well resolved and higher-level relationships are similar to those postulated in a previous study [45]. The tree provided strong support for most relationships within Sycophaginae. In all analyses, the subfamily was recovered as monophyletic with strong supports. With the exception of *Apocryptophagus*, which was paraphyletic with respect to *Sycophaga*, all genera were recovered as monophyletic with strong support.

Sycophaginae were divided in three well-supported clades: (i) the Australasian *Eukoebelea*; (ii) a clade comprising the Australasian *Pseudidarnes*, the Neotropical *Anidarnes *and the Oriental *Conidarnes *and (iii) a clade clustering *Apocryptophagus *and *Sycophaga *from the Old World and the Neotropical *Idarnes*. In all analyses, *Eukoebelea *was sister to all other Sycophaginae (PP 1.0, BP 99) and *Pseudidarnes *was basal to *Anidarnes*+*Conidarnes *(PP 0.99, BP 100).

Within the genus *Idarnes*, all three recognized species groups (namely *incerta*, *flavicollis *and *carme*) were recovered as monophyletic with strong support (PP 1.00, BP 100). The *Idarnes carme *group was basal to a clade clustering the *Idarnes incerta *and *flavicollis *groups (PP 1.00, BP 100).

Within the *Apocryptophagus */*Sycophaga *clade, the internodes were short, making the recovery of unambiguous phylogenetic information difficult. The basal node was a polytomy of four groups: (i) *Apocryptophagus *species associated with section *Sycocarpus*; (ii and iii) two continental Asiatic species that are respectively associated with *F. oligodon *and *F. orthoneura *and (iv) all the remaining *Apocryptophagus *and all *Sycophaga *species. The first group was recovered basal to the other 3 groups but this relationship was only supported in the Bayesian reconstruction (PP 0.96, BP 65). In the fourth group, the remaining *Apocryptophagus *and the *Sycophaga *species were distributed in five well-supported clades with no firmly established order of branching: (i) the *Apocryptophagus *species associated with *Adenosperma *fig trees (PP 1.00, BP 100); (ii) one *Apocryptophagus *species associated with *F. prostata*; (iii) a group including *A. agraensis *and *A. spinitarsus*, two cleptoparasites associated with *F. racemosa *and *F. variegata *respectively; (iv) a small group of *Apocryptophagus *species associated with *F. variegata *and *F. nodosa*; (v) a large and well supported clade (PP 0.99, BP 99) of *Apocryptophagus *and *Sycophaga *species exclusively associated with monoecious *Sycomorus *fig trees. Clade (v) was further subdivided into four groups: (i) the Afrotropical *Apocryptophagus *species with extremely long ovipositors associated with *F. sur*, *F. vallis-choudae *and *F. sycomorus*; (ii) an *Apocryptophagus *species with a long ovipositor associated with *F. sur*; (iii) a well supported clade grouping two species of early gallers, namely *A. gigas *associated with *F. sycomorus *and *F. mucuso *and *A. stratheni *associated with *F. racemosa*; (iv) a polytomy of three groups comprising *A. testacea *and *A. fusca *which were sister species; a well supported clade including all *Sycophaga *species; and a well supported and fully resolved clade grouping all species of *Apocryptophagus *from Madagascar and the Mascarene islands.

### Evolution of life history strategies

**Figure 6:**
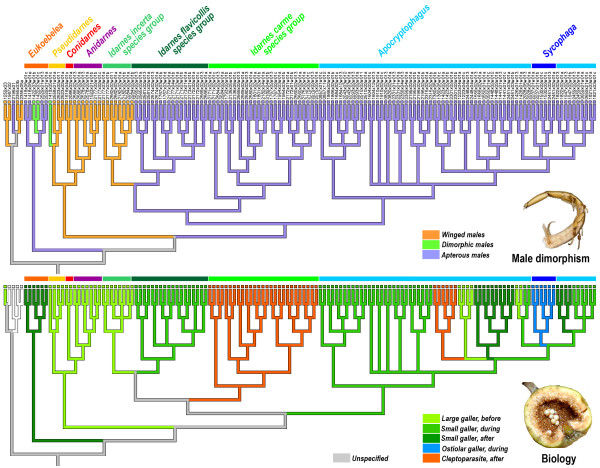
**Patterns of evolution of galling habits and male morphology**. Branch color reflects the most parsimonious ancestral area for that branch. Character states with significant proportion of total likelihood are indicated at the main nodes (likelihood threshold = 2.0). Reconstructions were performed using the ML-topology.

For the two studied traits (galling habits/brood size and male polymorphism), the character states of the ancestor of Sycophaginae remain ambiguous in both parsimony and likelihood reconstructions (Figure 6). The likelihood difference between winged and unwinged morphs of males is not significant (proportional likelihoods of 0.84 and 0.15, respectively). Whatever the ancestral state, aptery and wingedness evolved several times independently.

The ancestral biology of Sycophaginae is also ambiguous. Large gall-inducers, laying eggs in the syconium before pollination, medium-sized gall-inducers ovipositing during pollination or late gall-inducers ovipositing after pollination were equiprobable (proportional likelihoods of 0.21, 0.39 and 0.18, respectively). The ancestor of the clade *Idarnes *+ *Apocryptophagus/Sycophaga *was a medium sized gall-inducer that oviposited from outside at the same time as the pollinator (proportional likelihood of 0.87). Entrance through the ostiole appeared once and the ancestor was a medium-sized gall-inducer ovipositing from the outside during pollination (proportional likelihoods of 0.85). Cleptoparasitism appeared independently in the *Idarnes carme *group and in the genus *Apocryptophagus*. In both cases this new biology evolved from medium-sized gallers that oviposit from the outside of the syconium during pollination (proportional likelihoods of 0.86 and 0.95 respectively). The ability to develop large galls before pollination, whatever the ancestral biology, appeared at least four times independently (twice within *Apocryptophagus*) illustrating the lability of such biology.

## Discussion

### Sycophaginae phylogeny

We employed multiple genetic loci, extensive taxon sampling, and several different analytical approaches to reconstruct the evolutionary history of the Sycophaginae. The resulting topology is well resolved and provides strong support for most notable relationships within the subfamily. Our phylogeny generally agrees with a recent molecular study using more limited taxon sampling [45]. Therefore, we propose to lay the foundation for a revised classification of the subfamily.

Our analyses highlight three main clades that could be treated as tribes: (i) the Australasian *Eukoebelea*; (ii) the Australasian *Pseudidarnes*, the Neotropical *Anidarnes *and the Oriental *Conidarnes *and (iii) the Old World *Apocryptophagus *and *Sycophaga*, and the Neotropical *Idarnes*.

Our phylogeny strongly suggests *Eukoebelea *as the basal taxon within Sycophaginae. This is corroborated by several morphological characters: 1) the linear notauli without transverse crenulation (Figure 1A), 2) the absence of a well-delimitated supraclypeal area (Figure 1K), 3) both palpi (maxillary and labial) one-segmented, 4) the propodeal spiracles separated from the fore margin of the propodeum (Figure 1A) and 5) a long pronotum.

The phylogenetic reconstruction gives strong support for a *Pseudidarnes + Anidarnes + Conidarnes *clade, an alliance that has never been proposed by any of the previous taxonomic studies (but see [45]). This group is however well defined and characterized by 1) the deep and transversely crenulated notauli, axillular and frenal grooves (Figure 1D), all of them conspicuous; 2) the antennae inserted high on the face (Figure 1H); 3) the dorsellum conspicuous (Figure 1D); 4) the clypeal margin bilobed (Figure 1H).

*Idarnes *and *Apocryptophagus*/*Sycophaga *are sister taxa. This clade is difficult to define morphologically due to the morphological adaptations exhibited by the ostiolar *Sycophaga *and by the morphological characters of the *Idarnes incerta *species group. However, the group is characterized by at least some of the following characters: 1) short pronotum (except in ostiolar *Sycophaga*) (Figure 1B), 2) notauli deep and transversely crenulated (Figure 1B) (except in ostiolar *Sycophaga*, Figure 1E), 3) furcal pit enclosed within the mesosternum, 4) the clypeal margin straight or emarginated (Figure 1G) and 5) labial palpi two-segmented.

*Anidarnes *and *Idarnes *are never recovered in a single clade, which means that communities associated with Neotropical fig trees comprise two unrelated genera of Sycophaginae. Therefore, Sycophaginae communities associated with *Americana *fig trees in South America could be the result of two independent colonisations (see [45]).

*Anidarnes *is recovered as monophyletic. However, the inference of intra generic relationships requires more extensive sampling. *Idarnes *is monophyletic and the *incerta *group is deeply nested within the genus. Consequently and despite clear morphological differentiation from the two other species groups that share more characters, the *incerta *group does not represent a distinct genus as suggested by Rasplus and Soldati [43].

Females of *Sycophaga *are strongly differentiated and exhibit several apomorphies, such as a flattened and elongated head (Figure 1J), a short fore tibiae bearing teeth (Figure 1C), numerous spurs on the hind tibia (Figure 1F) and an absence of any sculpture on the mesosoma (Figure 1E). All these characters probably evolved as adaptations for crawling through the ostiole and make them easy to identify. A phylogeny based on morphological characters of females would probably support *Sycophaga *and *Apocryptophagus *as distinct lineages. However, in all our analyses, *Sycophaga *makes *Apocryptophagus *paraphyletic, with strong support. This result based on a molecular approach is corroborated by male morphology (Figure 1I). Indeed, males of *Apocryptophagus *and *Sycophaga *cannot be separated on tangible morphological characters. They exhibit a unique suite of synapomorphies (Figure 1): 1) the long peritremata of the abdominal spiracles (Figure 1I) that prevent the entry of water contained in the cavity of mature *Sycomorus *syconia into the tracheae and allow respiration [77,78], 2) the flat scape of the antenna and 3) the rectangular head (Figure 1I). Therefore, as already suggested by Bouček [33], we propose to consider *Apocryptophagus *as a junior synonym of *Sycophaga *(syn. nov). Hereafter, all *Apocryptophagus *species will be named under *Sycophaga*.

### Evolution of life history strategies

Our analyses show that the two investigated traits (galling habits/brood sizes and male polymorphism) are evolutionarily dynamic (Figure 6). Although the ancestral states for the whole subfamily cannot be firmly established, we highlight some interesting results.

Based on currently accepted biology of extant species, the ancestor of Sycophaginae was probably a galler. However, if *Eukoebelea *species were in fact cleptoparasites, as suggested in the introduction, then the feeding regime of the ancestor would be ambiguous. During sycophagine diversification, the same biologies re-evolved several times independently in distantly related lineages. Indeed, the ability to induce large galls evolved at least four times independently (*Anidarnes *+ *Pseudidarnes *+ *Conidarnes *clade, *Idarnes incerta *group and some *Apocryptophagus *species). Cleptoparastism evolved independently in *Idarnes *and *Apocryptophagus*. However, biological observations are missing for many sycophagine species and we cannot discard the possibility that cleptoparasitism also appeared in some other clades (four-five times if late gallers are in fact cleptoparasites). Clearly, more direct studies of larval ecology through the dissection of galls, observation of larval habits or experimental introductions and exclusions are needed. Life history strategies including whether sycophagine species are gallers or parasitic on other fig wasp larvae have only been demonstrated for few species based on field observations and experimental introductions (e.g. [50,52,54]. However, even careful experimental introductions and exclusions have limitations. For example, it is not possible to discriminate between species that oviposit early in the host galls and mostly feed on plant rather than insect tissue and wasps that oviposit later (once the gallmaker larva is fully developed) and mostly feed on insect tissue.

The ability to enter the syconia through the ostiole appeared only once (one lineage of *Sycophaga*, in our extended definition of the generic limits). Given that sycophagine fig wasps probably originated 50-40 Ma and following the *Sycophaga *stem group estimates from Cruaud *et al*. [45] (20-10 Ma), this biology took about 30 Ma to evolve. In the next 15 Ma, females evolved several morphological adaptations to crawl through the bracts that make their morphology strongly divergent from their sister lineages (see previous paragraph for details). It is noteworthy that one undescribed species of *Conidarnes *could also be an ostiolar gall-inducer. However, this hypothesis is based only on the external morphology of the species (JYR, pers. obs.) and needs to be confirmed by further field observations. Confirmation of this hypothesis would imply that the ability to enter the syconia appeared twice independently.

The evolutionary lability of male morphology is typical of traits that experience strong sexual selection, and has also been described for horns in scarabaeid beetles [79]. Within the sycophagines, winged and wingless morphs are distinguished not just by whether they have wings, but also by their behaviour (winged males can disperse outside the syconia whereas wingless males can fight and compete for females within the syconia) and important morphological differences. Winged males resemble their conspecific females closely, but wingless males are so divergent in form that they have repeatedly, and mistakenly, been classified in different taxa. They exhibit large mandibles, flattened head with small eyes and have fused mesosoma segments (Figures 4, 5). The occurrence of winged males, wingless males or both is highly labile across the phylogeny and closely related species can be monomorphic for opposite wing morphologies. For example, species in the *Idarnes incerta *group have winged males whereas all other *Idarnes *species produce apterous males. This polymorphism has also been observed in other groups such as aphids [80], ants [5] and parasitoid wasps [81] and suggests lability in the developmental processes leading to morph determination.

Our analyses indicate positive evolutionary correlations between large gall types/small brood sizes and winged males (*Anidarnes *+ *Pseudidarnes *+ *Conidarnes *clade and *Idarnes incerta *group all belonging to group 1 (Figure 6), a result that was previously reported for other NPFWs [61] and supports Hamilton's hypothesis [72]. *Pseudidarnes minerva *is the only exception known to date [61]. This Australian species associated with *Malvanthera *fig trees has both polymorphic males (wingless and winged morphs) and small broods. The wingless males do not fight, but dig a hole and and enter galls containing females to mate. This peculiar behaviour could explain why male polymorphism is retained in this species. In contrast, our analyses reveal that species with large brood size tend to produce wingless males.

We could not ascertain the ancestral morph. The proportional likelihood of aptery (0.84) is higher than the likelihood of the winged condition (0.15) but the difference is not significant. Therefore two scenarios can be proposed: 1) a basal loss of winged morphs and their later re-evolution and 2) a winged ancestor and the independent and recurrent losses of the winged morph in several lineages. In both cases, winged or wingless morph reappearance would be linked with galling strategy. It is noteworthy that the few *Apocryptophagus *species that produce large galls long before the pollinators (e.g. *A. gigas *and *A. stratheni*, some species associated with *Sycomorus *fig trees) exhibit wingless males only. These species have not re-evolved winged morphs even at very low densities [60]. This interesting result could be explained by other selective pressures that strongly counterselect the re-appearance of winged morphs. In particular, the presence of liquid in the syconia could explain why the males remain apterous. Indeed, in fig trees belonging to the *Sycomorus *subgenus, the cavity of the syconia can be filled with watery liquid during the interforal and male phases of the syconium development. Consequently, males emerge from their galls at a time when the galls are still covered by a thin film of liquid and literally swim between the galls during their search for mates [77]. Complementary explanations may include the large size of *Sycomorus *syconia in terms of numbers of flowers compared to that of other species groups hosting Sycophaginae. This could allow a higher number of large galls to develop within a single syconium [82], increasing the brood size of the species.

## Conclusions

The resulting trees from our phylogenetic analyses are mostly well resolved and highly supported. Therefore, we provide here an accurate phylogenetic framework that can be used in comparative evolutionary and ecological studies using Sycophaginae as a model system. Additional work is still needed on Sycophaginae phylogenetics. Indeed, our sampling represents only 10% of the estimated biodiversity of the group. Moreover, the relationships within *Sycophaga *(in our extended definition of the generic limits) are still somewhat uncertain. We also show that life history strategies of Sycophaginae are evolutionary labile, such that distantly related taxa share similar galling habits and vice-versa. The reconstruction of patterns of evolution of male polymorphism between or within the Sycophaginae genera suggests a lability in the developmental processes underlying the male morphology determination. Moreover, we show that winged males are favored in species that induce few galls within the syconia and wingless males are favored in species with large brood sizes. However, our results have to be taken with caution given that our inferences are based on a few number of studies dealing with sycophagine biology. Some species appear exceptional in that they apparently induce few galls but exhibit apterous males, which we hypothesize could be due to other factors that strongly select against the re-appearance of winged morphs (presence of liquid in the syconium, large number of flowers, etc). Untangling the full diversity of the biology of Sycophaginae will involve a tremendous amount of field work. What we have put forward here are a series of propositions on what is the biology of the species and how it evolved. Though preliminary in nature and although some propositions might be challenged by future work, this is the first proposal on how the biology of a group of non pollinating fig wasps diversified. Even within the current, limited, state of knowledge, we can firmly claim that we observe surprisingly abundant cases of repeated independent evolution of similar biologies. The Sycophaginae thus constitute an interesting model to test predictions on what factors will canalize the evolution of particular biologies and morphs.

## Methods

### Taxonomic and gene sampling

We expanded the sampling of our previous study [45] and included 73 ingroup species, 93% of which were represented by two individuals. All known genera of Sycophaginae as well as most species-groups were represented totaling about 1.5 times the number of described species (Additional file 1 Table S1, Figure 2). As phylogenetic relationships within Chalcidoidea are still unresolved, closer and more distant relatives were included as outgroups [83]. Five species belonging to the genera *Ceratosolen *(Agaonidae), *Odontofroggatia *(Epichrysomallinae), *Ficomila *(Eurytomidae) and *Megastigmus *(Torymidae) were used. All material was collected alive and fixed in 95% ethanol. Each time destructive extraction was used, vouchers were selected among specimens sampled from the same fig tree and the same syconium after carefull identification. Vouchers are deposited at CBGP, Montferrier-sur-Lez, France. A high definition image library of vouchers was also constructed, using EntoVision Premium Portable Imaging System, to allow future identification of specific taxa and traceability of our results (see Figures 4, 5 for examples). In the present study we combined one nuclear protein-coding gene (F2 copy of *elongation factor-1a, EF-1a*), two mitochondrial protein-coding genes (*Cytochrome oxydase I*, *COI *and *Cytochrome b*, *Cytb*) and *28S rRNA *(D2-D3 and D4-D5 expansion regions). Extraction, PCR and sequencing protocols follow [45]. Both strands for each overlapping fragment were assembled using the sequence editing software Geneious v3.7 [84]. All the sequences were deposited in GenBank (Additional file 1, Table S1).

### Phylogenetic analyses

Protein-coding genes (*COI*, *Cytb, EF) *were aligned using ClustalW 1.81 [85] with default gap opening, extension and substitution costs. For confirmation, alignments were translated to amino acids using MEGA version 4 [86]. Alignment of sequences encoding rRNA was based on secondary structure models [87,88] using the terminology developed by Kjer [89] and Gillespie *et al*.[90]. The structural model of rRNA fragments and alignment details follow Cruaud *et al*. [91].

Because our dataset comprised protein-coding genes and rRNA, we performed partitioned analyses implementing separate nucleotide substitution models for subsets of the data more likely to have experienced similar evolutionary processes (mitochondrial genes, *EF *and rRNA stems and loops). Best fitting model for each partition was identified using the Akaike information criterion [92] as implemented in MrAIC.pl 1.4.3[93].

Phylogenetic trees were estimated using maximum likelihood (ML) and Bayesian methods and all the analyses were conducted on a 150 cores Linux Cluster at CBGP.

We performed ML analyses and associated bootstrapping using the MPI-parallelized RAxML 7.0.4. [94]. GTRCAT approximation of models was used for ML boostrapping [94] (1000 replicates). Bootstrap percentage (BP) > 95% was considered as strong support and a BP < 70% as weak.

Bayesian analyses were conducted using a parallel version of MrBayes v. 3.1.1. (Huelsenbeck & Ronquist, 2001). We assumed across-partition heterogeneity in model parameters by considering the parameter m. Parameter values for the model were initiated with default uniform priors and branch lengths were estimated using default exponential priors. To improve mixing of the cold chain and avoid it converging on local optima, we used Metropolis-coupled Markov chain Monte Carlo (MCMC), with each run including a cold chain and three incrementally heated chains. The heating parameter was set to 0.02 in order to allow swap frequencies from 20% to 70%. We ran two independent runs of 10 million generations. All values were sampled every 1000 generations. For the initial determination of burn-in, we examined the plot of overall model likelihood against generation number to find the point where the likelihood started to fluctuate around a constant value. The points sampled prior to convergence of the chains were then discarded. We used a range of MCMC convergence and good mixing diagnostics following Cruaud *et al*.[91]. The results were based on the pooled samples from the stationary phases of the two independent runs. Posterior probabilities (PP) > 0.95 were considered as strong support.

### Evolution of life-history strategies

We compiled information on galling habits/brood size and male polymorphism of each sampled species from several literature sources [50-54,60,61,63,65,73-76] and from few unpublished observations made by us. We considered three different states for male polymorphism: 0: winged; 1: dimorphic (both winged and wingless males); 2: apterous. We used five different states to describe the biology of the Sycophaginae: 0: large gallers laying eggs in the syconium before pollination; 1: small-sized gallers ovipositing during pollination; 2: small-sized gallers ovipositing after pollination (late "gall-inducers"); 3: ostiolar galler ovipositing during the pollination and 4: cleptoparasites ovipositing long after pollination.

To infer the evolution of life-history strategies, we conducted Maximum Parsimony and ML ancestral state reconstructions using Mesquite 2.73 [95]. All reconstructions were performed on the ML topology. ML reconstructions were conducted using a single-rate Mk likelihood model for discrete morphological characters [96], which assumes that any particular change is equally probable. ML takes branch lengths into account and allows quantifying the uncertainty associated with each reconstructed ancestral state [97]. The likelihood decision threshold, i.e. the minimum difference in likelihood between the best and the next best state needed for assigning a state to a node was set to 2.0 [97].

## Authors' contributions

AC, CK and JYR designed the research. All the authors provided material or data. SvN, RU, RASP and JYR identified the wasps. FK and JYR identified the *Ficus *species. GG and RJZ performed and coordinated fig wasp DNA sequencing. AC and JYR performed the analyses. AC and JYR wrote the manuscript, with major comments from FK, PEH and CK. All authors read and approved the final manuscript.

## Supplementary Material

Additional file 1 **Table S1. List of Sycophaginae and outgroup species included in this study**. Voucher numbers, taxonomic information, host *Ficus *species, locality data and GenBank accession numbers for each sequenced fragment. More information is available from the authors upon request. Click here for file
