# Supplementary material for: Phylogeny and evolution of life-history strategies in the Sycophaginae non-pollinating fig wasps (Hymenoptera, Chalcidoidea)
Source: BMC Evol Biol. 2011 Jun 22;11:178. doi: 10.1186/1471-2148-11-178 (PMC3145598; doi:10.1186/1471-2148-11-178)
Supplement: Additional file 1 — Table S1. List of Sycophaginae and outgroup species included in this study. Voucher numbers, taxonomic information, host Ficus species, locality data and GenBank accession numbers for each sequenced fragment. More information is available from the authors upon request. [file 1471-2148-11-178-S1.DOC]

**Additional file. List of Sycophaginae and outgroup species included in this study.**

Voucher numbers, taxonomic information, host *Ficus* species, locality data and GenBank accession numbers for each sequenced fragment. More information is available from the authors upon request.

| **Voucher** | **Genus** | **Species** | **Host Ficus species** | **Countries** | ***COI*** | ***Cytb*** | ***EF-1a*** | **rRNA 28S** |
| --- | --- | --- | --- | --- | --- | --- | --- | --- |
| 0550_01w01a | *Apocryptophagus* | *comptoni* | *sycomorus* | Tanzania | HM770654 | ∅ | HM770497 | HM770716 |
| 0550_01w01c | *Apocryptophagus* | *comptoni* | *sycomorus* | Tanzania | JN001506 | ∅ | JN001645 | HM770716 |
| 0659_01w01a | *Apocryptophagus* | *explorator* | *mauritiana* | La Réunion | JN001507 | ∅ | JN001646 | HM770717 |
| 0659_21w01x | *Apocryptophagus* | *explorator* | *mauritiana* | La Réunion | HM770655 | HM770556 | HM770498 | HM770717 |
| 0820_02w013 | *Apocryptophagus* | sp. | *prostrata* | China | JN001508 | ∅ | JN001647 | HM770718 |
| 0820_02w01a | *Apocryptophagus* | sp. | *prostrata* | China | HM770656 | ∅ | HM770499 | HM770718 |
| 0857_01w01c | *Apocryptophagus* | sp. | *squamosa* | China | JN001509 | JN001585 | JN001648 | HM770719 |
| 0857_11w012 | *Apocryptophagus* | sp. | *squamosa* | China | HM770657 | HM770557 | HM770500 | HM770719 |
| 1223_04w01a | *Apocryptophagus* | *stratheni* | *racemosa* | India | HM770658 | HM770558 | HM770501 | HM770720 |
| 1223_04w01c | *Apocryptophagus* | *stratheni* | *racemosa* | India | JN001510 | ∅ | JN001649 | HM770720 |
| 1259_03 | *Megastigmus* | sp. | *virgata sessilis* | New Caledonia | GQ367876 | GQ367971 | HM770502 | GQ367582 |
| 1360_05w01b | *Apocryptophagus* | *agraensis* | *racemosa* | India | ∅ | JN001586 | JN001650 | HM770664 |
| 1360_05w01c | *Apocryptophagus* | *agraensis* | *racemosa* | India | HM770659 | HM770559 | HM770503 | HM770664 |
| 1418_05w01x | *Pseudidarnes* | *minerva* | *rubiginosa* | Australia | HM770660 | HM770560 | HM770504 | HM770665 |
| 1418_06w01c | *Eukoebelea* | sp. | *rubiginosa* | Australia | JN001511 | JN001587 | JN001651 | HM770666 |
| 1418_06w01e | *Eukoebelea* | sp. | *rubiginosa* | Australia | HM770661 | HM770561 | HM770505 | HM770666 |
| 1422_03w01b | *Eukoebelea* | sp. | *obliqua* | Australia | JN001512 | ∅ | JN001652 | HM770667 |
| 1422_03w01c | *Eukoebelea* | sp. | *obliqua* | Australia | HM770662 | ∅ | HM770506 | HM770667 |
| 1423_02w01a | *Apocryptophagus* | *spinitarsus* | *variegata* | Australia | JN001513 | JN001588 | JN001653 | JN001490 |
| 1423_02w01b | *Apocryptophagus* | *spinitarsus* | *variegata* | Australia | JN001514 | JN001589 | JN001654 | JN001490 |
| 1426_01w013 | *Apocryptophagus* | *testacea* | *racemosa* | Australia | HM770663 | HM770562 | HM770507 | HM770668 |
| 1426_01w01a | *Apocryptophagus* | *testacea* | *racemosa* | Australia | JN001515 | JN001590 | JN001655 | HM770668 |
| 1441_01w013 | *Apocryptophagus* | sp. | *sur* | Principe | ∅ | JN001591 | ∅ | HM770669 |
| 1441_01w01c | *Apocryptophagus* | sp. | *sur* | Principe | HM770607 | HM770563 | HM770508 | HM770669 |
| 1448_02w012 | *Apocryptophagus* | *sycomori* | *sycomorus* | Madagascar | JN001516 | JN001592 | JN001656 | JN001491 |
| 1448_02w01a | *Apocryptophagus* | *sycomori* | *sycomorus* | Madagascar | JN001517 | JN001593 | JN001657 | JN001491 |
| 1523_01w01b | *Anidarnes* | *bicolor* | *aurea* | USA | ∅ | ∅ | JN001658 | ∅ |
| 1532_02w01c | *Sycophaga* | *sycomori* | *sycomorus* | South Africa | HM770608 | HM770564 | HM770509 | HM770670 |
| 1532_02w01x | *Sycophaga* | *sycomori* | *sycomorus* | South Africa | JN001518 | ∅ | ∅ | HM770670 |
| 1556_02w013 | *Apocryptophagus* | sp. | *sur* | Guinea | JN001519 | JN001594 | ∅ | JN001492 |
| 1556_02w01a | *Apocryptophagus* | sp. | *sur* | Guinea | JN001520 | ∅ | ∅ | JN001492 |
| 1604_02w01x | *Apocryptophagus* | sp. | *orthoneura* | China | HM770609 | HM770565 | HM770510 | HM770671 |
| 1604_12w013 | *Apocryptophagus* | sp. | *orthoneura* | China | JN001521 | JN001595 | ∅ | HM770671 |
| 1616_04w01x | *Conidarnes* | sp. | *altissima* | China | JN001522 | JN001596 | JN001659 | JN001493 |
| 1684_02w01x | *Apocryptophagus* | sp. | *septica* | Taiwan | JN001523 | JN001597 | JN001660 | HM770672 |
| 1684_12w013 | *Apocryptophagus* | sp. | *septica* | Taiwan | HM770610 | HM770566 | HM770511 | HM770672 |
| 1767_02w013 | *Idarnes* | sp. | *amazonica* | French Guiana | HM770611 | HM770567 | HM770512 | HM770673 |
| 1767_02w01c | *Idarnes* | sp. | *amazonica* | French Guiana | JN001524 | JN001598 | JN001661 | HM770673 |
| 1767_03w01b | *Idarnes* | sp. | *amazonica* | French Guiana | JN001525 | JN001599 | JN001662 | HM770674 |
| 1767_03w01d | *Idarnes* | sp. | *amazonica* | French Guiana | HM770612 | HM770568 | HM770513 | HM770674 |
| 1793_02w01b | *Idarnes* | sp. | *petiolaris* | Mexico | ∅ | JN001600 | JN001663 | ∅ |
| 1801_02w01a | *Idarnes* | sp. | *obtusifolia* | Mexico | HM770613 | HM770569 | HM770514 | HM770675 |
| 1801_02w01b | *Idarnes* | sp. | *obtusifolia* | Mexico | JN001526 | JN001601 | JN001664 | HM770675 |
| 1821_03w01a | *Apocryptophagus* | *randrianjohanyi* | *trichoclada* | Madagascar | JN001527 | ∅ | JN001665 | HM770676 |
| 1821_03w01c | *Apocryptophagus* | *randrianjohanyi* | *trichoclada* | Madagascar | HM770614 | HM770570 | HM770515 | HM770676 |
| 1822_02w013 | *Apocryptophagus* | *labati* | *botryoides* | Madagascar | JN001528 | JN001602 | JN001666 | HM770677 |
| 1822_02w01a | *Apocryptophagus* | *labati* | *botryoides* | Madagascar | HM770615 | HM770571 | HM770516 | HM770677 |
| 1866_02w01a | *Apocryptophagus* | sp. | *lepicarpa* | Malaysia | HM770616 | HM770572 | HM770517 | HM770678 |
| 1866_02w01b | *Apocryptophagus* | sp. | *lepicarpa* | Malaysia | JN001529 | ∅ | JN001667 | HM770678 |
| 1910_02w01a | *Apocryptophagus* | sp. | *oligodon* | China | HM770617 | HM770573 | HM770518 | HM770679 |
| 1910_02w01c | *Apocryptophagus* | sp. | *oligodon* | China | JN001530 | JN001603 | JN001668 | HM770679 |
| 1947_02w013 | *Apocryptophagus* | *nesiotes* | *sakalavarum* | Madagascar | JN001531 | JN001604 | JN001669 | HM770680 |
| 1947_02w01b | *Apocryptophagus* | *nesiotes* | *sakalavarum* | Madagascar | HM770618 | HM770574 | HM770519 | HM770680 |
| 1954_03w01a | *Idarnes* | sp. | *citrifolia* | Costa Rica | JN001532 | JN001605 | JN001670 | JN001494 |
| 1954_03w01b | *Idarnes* | sp. | *citrifolia* | Costa Rica | JN001533 | ∅ | JN001671 | JN001494 |
| 1987_02 | *Ficomila* | sp. | *variegata* | Malaysia | GQ367946 | GQ368043 | HM770520 | GQ367656 |
| 2028_05w013 | *Apocryptophagus* | *fusca* | *racemosa* | Indonesia | JN001534 | JN001606 | JN001672 | HM770681 |
| 2028_05w01a | *Apocryptophagus* | *fusca* | *racemosa* | Indonesia | HM770619 | HM770575 | HM770521 | HM770681 |
| 2043_03w012 | *Apocryptophagus* | sp. | *nr fistulosa* | Indonesia | JN001535 | JN001607 | JN001673 | JN001495 |
| 2043_03w01a | *Apocryptophagus* | sp. | *nr fistulosa* | Indonesia | JN001536 | JN001608 | JN001674 | JN001495 |
| 2085_02w01a | *Conidarnes* | sp. | *sumatrana* | Indonesia | HM770620 | HM770576 | HM770522 | HM770682 |
| 2136_05w01b | *Idarnes* | sp. | *eximia* | Brazil | HM770621 | HM770577 | HM770523 | HM770683 |
| 2136_05w01c | *Idarnes* | sp. | *eximia* | Brazil | JN001537 | ∅ | ∅ | HM770683 |
| 2171_02w01a | *Idarnes* | sp. | *trachelosyce* | Costa Rica | HM770622 | HM770578 | HM770524 | HM770684 |
| 2171_02w01c | *Idarnes* | sp. | *trachelosyce* | Costa Rica | JN001538 | JN001609 | JN001675 | HM770684 |
| 2171_03w01a | *Idarnes* | sp. | *trachelosyce* | Costa Rica | JN001539 | ∅ | JN001676 | HM770685 |
| 2171_03w01b | *Idarnes* | sp. | *trachelosyce* | Costa Rica | HM770623 | ∅ | HM770525 | HM770685 |
| 2172_01w01a | *Idarnes* | sp. | *poponei* | Costa Rica | JN001540 | JN001610 | JN001677 | JN001496 |
| 2172_01w01b | *Idarnes* | sp. | *poponei* | Costa Rica | JN001541 | JN001611 | JN001678 | JN001496 |
| 2172_02w01a | *Idarnes* | sp. | *poponei* | Costa Rica | JN001542 | ∅ | JN001679 | JN001497 |
| 2172_02w01c | *Idarnes* | sp. | *poponei* | Costa Rica | JN001543 | ∅ | JN001680 | JN001497 |
| 2173_01w01a | *Idarnes* | sp. | *obtusifolia* | Costa Rica | JN001544 | JN001612 | JN001681 | JN001498 |
| 2173_01w01b | *Idarnes* | sp. | *obtusifolia* | Costa Rica | JN001545 | JN001613 | JN001682 | JN001498 |
| 2175_02w01a | *Idarnes* | sp. | *jimenezii* | Costa Rica | JN001546 | JN001614 | JN001683 | JN001499 |
| 2175_02w01c | *Idarnes* | sp. | *jimenezii* | Costa Rica | JN001547 | JN001615 | JN001684 | JN001499 |
| 2177_02w01b | *Anidarnes* | sp. | *perforata* | Costa Rica | HM770624 | ∅ | HM770526 | HM770686 |
| 2177_02w01c | *Anidarnes* | sp. | *perforata* | Costa Rica | JN001548 | ∅ | JN001685 | HM770686 |
| 2177_03w01a | *Idarnes* | sp. | *perforata* | Costa Rica | HM770625 | HM770579 | HM770527 | HM770687 |
| 2177_03w01b | *Idarnes* | sp. | *perforata* | Costa Rica | JN001549 | JN001616 | JN001686 | HM770687 |
| 2177_04w01a | *Idarnes* | sp. | *perforata* | Costa Rica | JN001550 | JN001617 | JN001687 | JN001500 |
| 2177_04w01b | *Idarnes* | sp. | *perforata* | Costa Rica | JN001551 | JN001618 | JN001688 | JN001500 |
| 2182_02w01a | *Idarnes* | sp. | *goldmanii* | Costa Rica | HM770626 | HM770580 | HM770528 | HM770688 |
| 2182_02w01b | *Idarnes* | sp. | *goldmanii* | Costa Rica | JN001552 | JN001619 | JN001689 | HM770688 |
| 2182_04w01a | *Idarnes* | sp. | *goldmanii* | Costa Rica | JN001553 | JN001620 | JN001690 | JN001501 |
| 2182_04w01b | *Idarnes* | sp. | *goldmanii* | Costa Rica | JN001554 | JN001621 | JN001691 | JN001501 |
| 2195_02w01a | *Apocryptophagus* | sp. | *subcuneata* | Indonesia | HM770627 | HM770581 | HM770529 | HM770689 |
| 2195_02w01b | *Apocryptophagus* | sp. | *subcuneata* | Indonesia | JN001555 | JN001622 | JN001692 | HM770689 |
| 2196_01 | *Ceratosolen* | sp. | *comitis* | Indonesia | GQ367958 | GQ368053 | HM770530 | GQ367670 |
| 2196_02w01a | *Apocryptophagus* | sp. | *comitis* | Indonesia | JN001556 | JN001623 | JN001693 | JN001502 |
| 2196_02w01b | *Apocryptophagus* | sp. | *comitis* | Indonesia | JN001557 | JN001624 | JN001694 | JN001502 |
| 2237_01w01a | *Sycophaga* | *cyclostigma* | *sur* | Gabon | HM770628 | HM770582 | HM770531 | HM770690 |
| 2237_01w01b | *Sycophaga* | *cyclostigma* | *sur* | Gabon | JN001558 | JN001625 | JN001695 | HM770690 |
| 2284_02w01a | *Idarnes* | sp. | *hemsleyana* | Costa Rica | JN001559 | ∅ | JN001696 | JN001503 |
| 2284_02w01b | *Idarnes* | sp. | *hemsleyana* | Costa Rica | JN001560 | JN001626 | JN001697 | JN001503 |
| 2293_02w013 | *Apocryptophagus* | sp. | *congesta* | Indonesia | JN001561 | JN001627 | JN001698 | HM770691 |
| 2293_02w01a | *Apocryptophagus* | sp. | *congesta* | Indonesia | HM770629 | HM770583 | HM770532 | HM770691 |
| 2301_02w01a | *Apocryptophagus* | sp. | *variegata* | Indonesia | HM770630 | HM770584 | HM770533 | HM770692 |
| 2301_02w01b | *Apocryptophagus* | sp. | *variegata* | Indonesia | JN001562 | JN001628 | JN001699 | HM770692 |
| 2315_02w01a | *Apocryptophagus* | sp. | *mollior* | Indonesia | HM770631 | HM770585 | HM770534 | HM770693 |
| 2315_02w01b | *Apocryptophagus* | sp. | *mollior* | Indonesia | JN001563 | JN001629 | JN001700 | HM770693 |
| 2331_01w013 | *Apocryptophagus* | sp. | *variegata* | Indonesia | JN001564 | JN001630 | JN001701 | HM770694 |
| 2331_01w01a | *Apocryptophagus* | sp. | *variegata* | Indonesia | HM770632 | HM770586 | HM770535 | HM770694 |
| 2355_03b | *Odontofroggattia* | sp. | *microcarpa* | Indonesia | HM770633 | HM770587 | HM770536 | HM770695 |
| 2355_05a | *Odontofroggattia* | *ishii* | *microcarpa* | Indonesia | HM770634 | HM770588 | HM770537 | HM770696 |
| 2356_04w013 | *Apocryptophagus* | sp. | *nodosa* | Indonesia | HM770635 | HM770589 | HM770538 | HM770697 |
| 2356_04w01a | *Apocryptophagus* | sp. | *nodosa* | Indonesia | JN001565 | JN001631 | JN001702 | HM770697 |
| 2448_04w013 | *Apocryptophagus* | *gigas* | *sycomorus* | Senegal | JN001566 | ∅ | JN001703 | HM770698 |
| 2448_04w01a | *Apocryptophagus* | *gigas* | *sycomorus* | Senegal | HM770636 | HM770590 | HM770539 | HM770698 |
| 2451_02w013 | *Sycophaga* | *silvestrii* | *sur* | Senegal | JN001567 | JN001632 | JN001704 | JN001504 |
| 2451_02w01a | *Sycophaga* | *silvestrii* | *sur* | Senegal | JN001568 | JN001633 | JN001705 | JN001504 |
| 2451_03w01a | *Apocryptophagus* | sp. | *sur* | Senegal | HM770637 | HM770591 | HM770540 | HM770699 |
| 2451_03w01b | *Apocryptophagus* | sp. | *sur* | Senegal | JN001569 | JN001634 | JN001706 | HM770699 |
| 2459_02w013 | *Apocryptophagus* | sp. | *tiliifolia* | Madagascar | JN001570 | JN001635 | JN001707 | HM770700 |
| 2459_02w01a | *Apocryptophagus* | sp. | *tiliifolia* | Madagascar | HM770638 | HM770592 | HM770541 | HM770700 |
| 2510_02w011 | *Apocryptophagus* | sp. | *dissipata* | Solomon Islands | JN001571 | JN001636 | JN001708 | HM770701 |
| 2510_02w01a | *Apocryptophagus* | sp. | *dissipata* | Solomon Islands | HM770639 | HM770593 | HM770542 | HM770701 |
| 2523_02w011 | *Pseudidarnes* | sp. | *baola* | Solomon Islands | HM770640 | HM770594 | HM770543 | HM770702 |
| 2523_02w01a | *Pseudidarnes* | sp. | *baola* | Solomon Islands | JN001572 | ∅ | ∅ | HM770702 |
| 2529_01w013 | *Eukoebelea* | sp. | *glandifera* | Solomon Islands | HM770641 | HM770595 | HM770544 | HM770703 |
| 2529_01w01a | *Eukoebelea* | sp. | *glandifera* | Solomon Islands | JN001573 | JN001637 | JN001709 | HM770703 |
| 2558_01w01a | *Pseudidarnes* | sp. | *obliqua* | Australia | HM770642 | HM770596 | HM770545 | HM770704 |
| 2560_02w013 | *Idarnes* | sp. | *citrifolia* | Brazil | JN001574 | JN001638 | JN001710 | JN001505 |
| 2560_02w01a | *Idarnes* | sp. | *citrifolia* | Brazil | JN001575 | ∅ | ∅ | JN001505 |
| 2562_02w011 | *Idarnes* | sp. | *citrifolia* | Brazil | HM770643 | ∅ | HM770546 | HM770705 |
| 2562_02w01a | *Idarnes* | sp. | *citrifolia* | Brazil | JN001576 | ∅ | JN001711 | HM770705 |
| 2565_02w013 | *Idarnes* | sp. | *eximia* | Brazil | HM770644 | HM770597 | HM770547 | HM770706 |
| 2565_02w01a | *Idarnes* | sp. | *eximia* | Brazil | ∅ | JN001639 | JN001712 | HM770706 |
| 2566_02w013 | *Idarnes* | sp. | *eximia* | Brazil | HM770645 | HM770598 | HM770548 | HM770707 |
| 2566_02w01a | *Idarnes* | sp. | *eximia* | Brazil | JN001577 | JN001640 | JN001713 | HM770707 |
| 2569_02w013 | *Idarnes* | sp. | *eximia* | Brazil | HM770646 | HM770599 | HM770549 | HM770708 |
| 2569_02w01a | *Idarnes* | sp. | *eximia* | Brazil | ∅ | JN001641 | ∅ | HM770708 |
| 2574_02w013 | *Idarnes* | sp. | *crocata* | Brazil | JN001578 | JN001642 | JN001714 | HM770709 |
| 2574_02w01a | *Idarnes* | sp. | *crocata* | Brazil | HM770647 | HM770600 | HM770550 | HM770709 |
| 2575_02w013 | *Idarnes* | sp. | *crocata* | Brazil | JN001579 | JN001643 | JN001715 | HM770710 |
| 2575_02w01a | *Idarnes* | sp. | *crocata* | Brazil | HM770648 | HM770601 | HM770551 | HM770710 |
| 2578_02w013 | *Anidarnes* | sp. | *crocata* | Brazil | JN001580 | ∅ | JN001716 | HM770711 |
| 2578_02w01a | *Anidarnes* | sp. | *crocata* | Brazil | HM770649 | HM770602 | HM770552 | HM770711 |
| 2580_02w013 | *Idarnes* | sp. | *obtusifolia* | Brazil | HM770650 | HM770603 | HM770553 | HM770712 |
| 2580_02w01a | *Idarnes* | sp. | *obtusifolia* | Brazil | JN001581 | JN001644 | JN001717 | HM770712 |
| 2581_02w013 | *Idarnes* | sp. | *obtusifolia* | Brazil | HM770651 | HM770604 | HM770554 | HM770713 |
| 2581_02w01a | *Idarnes* | sp. | *obtusifolia* | Brazil | JN001582 | ∅ | JN001718 | HM770713 |
| 2584_02w013 | *Idarnes* | sp. | *obtusifolia* | Brazil | HM770652 | HM770605 | HM770555 | HM770714 |
| 2584_02w01a | *Idarnes* | sp. | *obtusifolia* | Brazil | JN001583 | ∅ | JN001719 | HM770714 |
| 2586_02w013 | *Anidarnes* | sp. | *obtusifolia* | Brazil | JN001584 | ∅ | ∅ | HM770715 |
| 2586_02w01a | *Anidarnes* | sp. | *obtusifolia* | Brazil | HM770653 | HM770606 | ∅ | HM770715 |
